# Engineered microenvironments for synergistic VEGF – Integrin signalling during vascularization

**DOI:** 10.1016/j.biomaterials.2017.02.024

**Published:** 2017-05

**Authors:** Vladimíra Moulisová, Cristina Gonzalez-García, Marco Cantini, Aleixandre Rodrigo-Navarro, Jessica Weaver, Mercedes Costell, Roser Sabater i Serra, Matthew J. Dalby, Andrés J. García, Manuel Salmerón-Sánchez

**Affiliations:** a Division of Biomedical Engineering, School of Engineering, University of Glasgow, Glasgow G12 8LT, United Kingdom; b Departament de Bioquimica i Biologia Molecular, Universitat de València, Burjassot, Spain; c Centre for Biomaterials and Tissue Engineering, Universitat Politècnica de València, Spain; d Networking Research Center on Bioengineering, Biomaterials and Nanomedicine (CIBER-BBN), Valencia, Spain; e Centre for Cell Engineering, Institute of Molecular Cell and Systems Biology, University of Glasgow, Joseph Black Bld, University Avenue, Glasgow G12 8QQ, United Kingdom; f Woodruff School of Mechanical Engineering, Petit Institute for Bioengineering and Bioscience, Georgia Institute of Technology, Atlanta, GA 30332, USA

**Keywords:** Fibronectin, Protein assembly, Growth factors, VEGF, Vascularization, poly(ethyl acrylate)

## Abstract

We have engineered polymer-based microenvironments that promote vasculogenesis both *in vitro* and *in vivo* through synergistic integrin-growth factor receptor signalling. Poly(ethyl acrylate) (PEA) triggers spontaneous organization of fibronectin (FN) into nanonetworks which provide availability of critical binding domains. Importantly, the growth factor binding (FNIII_12-14_) and integrin binding (FNIII_9-10_) regions are simultaneously available on FN fibrils assembled on PEA. This material platform promotes synergistic integrin/VEGF signalling which is highly effective for vascularization events *in vitro* with low concentrations of VEGF. VEGF specifically binds to FN fibrils on PEA compared to control polymers (poly(methyl acrylate), PMA) where FN remains in a globular conformation and integrin/GF binding domains are not simultaneously available. The vasculogenic response of human endothelial cells seeded on these synergistic interfaces (VEGF bound to FN assembled on PEA) was significantly improved compared to soluble administration of VEGF at higher doses. Early onset of VEGF signalling (PLCγ1 phosphorylation) and both integrin and VEGF signalling (ERK1/2 phosphorylation) were increased only when VEGF was bound to FN nanonetworks on PEA, while soluble VEGF did not influence early signalling. Experiments with mutant FN molecules with impaired integrin binding site (FN-RGE) confirmed the role of the integrin binding site of FN on the vasculogenic response via combined integrin/VEGF signalling. *In vivo* experiments using 3D scaffolds coated with FN and VEGF implanted in the murine fat pad demonstrated pro-vascularization signalling by enhanced formation of new tissue inside scaffold pores. PEA-driven organization of FN promotes efficient presentation of VEGF to promote vascularization in regenerative medicine applications.

## Highlights

• VEGF specifically binds to spontaneously formed fibronectin assembled on polymers.
• Highly efficient VEGF presentation allows simultaneous VEGFR and integrins signalling.
• Low VEGF amount for endothelial cell reorganization towards primitive vasculature.
• Fibronectin and VEGF coated PEA scaffold stimulates vessel growth *in vivo*.

## Graphical abstract

**Figure:**
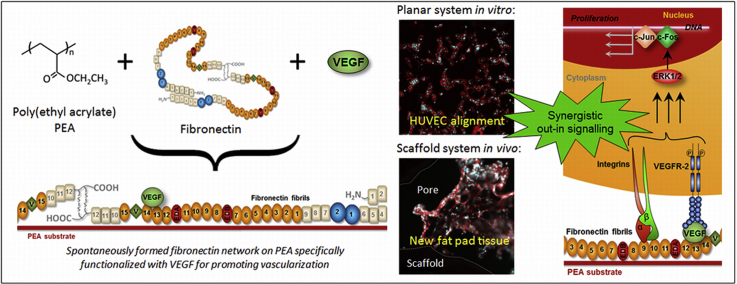

## 1 Introduction

Fully developed vascular networks are essential for cell growth and tissue formation as they aid nutrient and oxygen supply and removal of toxic metabolites [1]. Detailed understanding of the process of vessel formation can provide a powerful tool to control vascularization in pathological conditions, and it can prove useful in tissue engineering and regenerative medicine where developing functional vascularized tissue is still a major challenge [2].

New vessels can be formed in two distinct ways: either via sprouting of existing vessels (angiogenesis), or development of *de novo* vessels from progenitor cells (vasculogenesis). As these processes are complex, a dynamic interaction between cells, growth factors (GFs) and extracellular matrix (ECM) components involves strict temporal and spatial regulation to allow both a nascent tube formation and vessel maturation [3]. Angioblasts derived from bone marrow progenitor cells differentiate into endothelial cells and together with smooth muscle cells go on to form the vessel [3]. The presence of other cell types such as pericytes is important for vessel maturation [3]. A detailed role of secreted GFs and cytokines orchestrating this process has still not been fully elucidated, however a prominent role for the vascular endothelial GF (VEGF) family and their receptors and fibroblastic GF (FGF) has been repeatedly described [4], [5]. Additionally, the complex ECM structure consisting of proteins such as fibrin, fibronectin (FN), collagens, laminin and others serve not only as a mechanical scaffold for cell migration, proliferation and non-specific retention of GFs but create a dynamic environment able to bind GFs specifically, forming gradients and possibly releasing them on demand during proteolytic degradation [6], [7], [8], [9].

*In vitro*, there has been remarkable success in forming new vessels using various microfluidic chambers to study the fundamentals of vasculogenesis [10], [11]. However, it is challenging to translate these results to *in vivo* applications. Considerable research closer to eventual *in vivo* applications has focused on engineering different 3D environments (hydrogels, synthetic polymer matrices) containing key GFs such as VEGF, FGF, platelet-derived GF (PDGF) or insulin-like GF (IGF) and/or cell adhesion and ECM protein binding motifs that support endogenous endothelial cell growth [8], [12]. However, this approach generally requires quite high doses of GFs and faces issues such as low GF stability and accessibility. Strategies incorporating molecules able to sequester GFs are promising to present GFs more efficiently and considerably reduce GF doses needed for biological effects [12]. ECM proteins such as fibrinogen or FN, or their engineered fragments, have been utilized to bind and present bone morphogenetic protein 2 (BMP-2), PDGF, or VEGF, and investigated in their ability to promote wound healing [13], [14], [15].

**Fig. 1:**
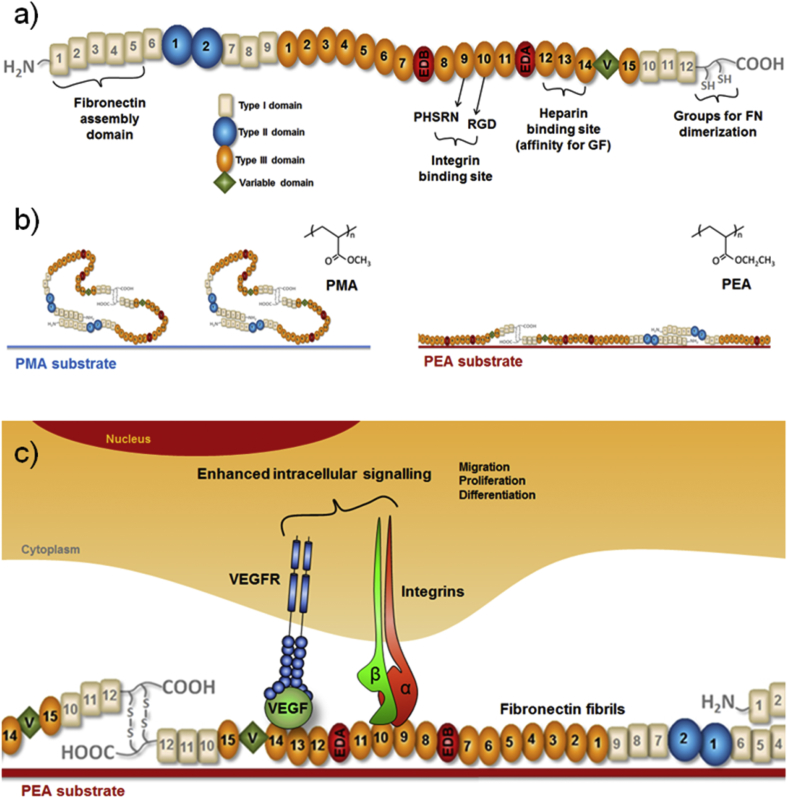
Integrin-VEGF synergistic signalling triggered by FN organized into nanonetworks on PEA. a) Fibronectin molecule with domains depicted; b) fibronectin assembly on two different polymer substrates; on PMA it remains in globular conformation, whereas on PEA, FN assembly is triggered and networks are assembled; c) scheme of synergistic effect of VEGF bound to FN on cell signalling: the presentation of VEGF bound to FN in close vicinity of integrin binding site effectively enhances outside-in signalling and allows to VEGFR and integrins to work in synergy.

FN is a large protein that is known to bind GFs from several families [16], [17], [18]. However, for this process to be efficient, the molecule needs to be unfolded to display GF binding regions [18]. Plasma FN exists as a dimer and consists of three types of modules, FNI, FNII and FNIII (Fig. 1a). Once secreted by cells, the globular conformation of the protein changes into a fibrillar one through an integrin-mediated process where cysteine-rich FNI modules facilitate FN-FN interaction and assembly of FN networks [19]. Cell adhesion peptide motifs such as RGD and its synergy sequence PHSRN in the central cell binding III_9-10_ domain interact with cells by binding integrins, whereas the heparin II binding domain in FNIII_12-14_ can bind different GFs [16], [17], [18]. Spontaneous FN fibrillogenesis has been described as FN absorbs onto PEA that recapitulates the natural organization of FN in the ECM [20], [21] and results in high availability of integrin and GF binding regions (Fig. 1b). Since the integrin binding region (FNIII_9-10_) and the GF binding region (FNIII_12-14_) are adjacent to each other, this organization of FN on PEA promotes crosstalk between integrins and GF receptors and then synergistic integrin and GF receptor signalling [22], [23]. In this study, FN is assembled into fibrillar nanonetworks on PEA to engineer synergistic VEGF-presenting microenvironments that promote vasculogenic responses in endothelial cells (Fig. 1c). This biomimetic system stimulates pro-angiogenic processes both *in vitro* and *in vivo* using ultra low doses of the GF, and thus represents a robust and safe strategy to improve vascularization in tissue engineering approaches.

## 2 Material and methods

### 2.1 Polymer surfaces and scaffolds

Poly(methyl acrylate) (PMA) and poly(ethyl acrylate) (PEA) sheets were prepared by radical polymerization of methyl acrylate and ethyl acrylate solutions using 1% benzoin as photoinitiator. Thin films were prepared by spin-coating PMA (6%) and PEA (2.5%) solutions in toluene on cleaned glass cover slips for 30 s at 3000 rpm and 2000 rpm, respectively. Before use, samples were oven dried at 60 °C and vacuum extracted.

PMA and PEA scaffolds were fabricated using a 3D-printed PVA template (Ikasia Technologies SL software). The scaffold template consisted of stacked layers of aligned fibres of 400 μm diameter, with a fibre-fibre distance of 300 μm. The fibres in a layer were perpendicularly oriented in respect to the previous one in order to create a mesh of PVA fibres that was used as a template for thermal polymerization of methyl or ethyl acrylate monomer. The reaction was carried out at 60 °C for 24 h using 1% benzoyl peroxide as initiatior and 2% ethylene glycol dimethacrylate as crosslinker. After the polymerization, the PVA template was dissolved in water and resulting scaffolds were washed several times in ethanol to remove all traces of PVA fibres, dried at vacuum, and cut in cylindrical disks of 5 mm diameter and 2 mm thickness.

### 2.2 Substrate functionalization with proteins

Spin-coated polymer samples were sterilized under UV for 20 min. Polymers were coated with a human plasma fibronectin (FN) solution (20 μg/ml, recombinant human VEGF 165, R&D Systems) for 1 h, washed with PBS, and incubated in VEGF (25 ng/ml, R&D) for 1 h. Control samples without either FN or VEGF were incubated in PBS for the same time as coated samples. Functionalized substrates were washed with PBS prior to further experiments. All incubations were done at room temperature.

To collect plasma FN carrying the D to E mutation in the RGD motif in FNIII_10_ module, we generated inducible Mx-Cre mice with heterozygous FN gene: one allele with floxed FN [24] and the other allele with the mutation FN^RGE^ [25]. After Cre induction in liver by 3 intraperitoneal injections of 250 μg polyinosinic-polycytidylic acid at 2-day intervals, blood was collected from FN^wt/wt^ and FN^flox/RGE^;Mx-Cre mice using 0.5 M EDTA as anticoagulant in non-heparinized capillaries, centrifuged at 3000 rpm for 20 min and the plasma FN was purified from the supernatant (plasma) using gelatin-Sepharose (GE Healthcare Life Sciences) affinity chromatography adapted to minicolumns (Poly-Prep, Bio-Rad). Briefly, the columns were washed with 0.5 NaCl in 10 mM Tris-HCl pH 7.4 and FN was eluted with 2 M urea in TBS (0.15 M NaCl in 10 mM Tris-HCl, pH 7.4) and dialyzed against TBS. Purified FN was analyzed by 8% SDS-PAGE and stained with Coomassie brilliant blue, and by Western blot. Wild-type and mutant FN containing RGE were used for the coating in the same way as the human purified FN (20 μg/ml).

FN was adsorbed overnight onto the scaffold from a protein solution (20 μg/ml) at 37 °C. Then, 1% bovine serum albumin (BSA) was adsorbed for 30 min at room temperature before exposure to VEGF in a PBS solution (10 μg/ml) for 1 h. Protein incubation was facilitated by vacuum allowing the solution to enter into the scaffold pores. Samples were rinsed in PBS to remove non-adsorbed proteins and kept in PBS until implantation.

### 2.3 Atomic force microscopy

Functionalized planar substrates were washed three times with ultrapure water followed by drying with nitrogen. Sample surfaces were imaged with atomic force microscope NanoWizard 3 (JPK) using a MPP cantilever (Bruker) with spring constant 3 N/m and resonance frequency of 75 kHz in tapping mode.

### 2.4 ELISA

VEGF bound to flat substrates was assayed indirectly by measuring remaining VEGF in coating solution with sandwich ELISA kit according to manufacturer's instructions (DuoSetDY293B, R&D Systems). Levels of phosphorylation of ERK1/2 in human umbilical vascular endothelial cells (HUVEC) seeded onto functionalized surfaces were assesses after 30 min incubation using DuoSet ELISA kit following manufacturer's instructions (DuoSetDYC1018B, R&D Systems).

### 2.5 Western blotting

Cells were lysed and processed under denaturing and reducing conditions. Then, proteins were transferred to PVDF membranes in a semidry blotter. After the transfer, membranes were blocked with 5% BSA in Tris-buffered saline with 0.1% Tween 20 (TBST) for one hour at RT, and then cut at the 70 kDa mark to allow probing for pPLCγ1/pFAK and α-tubulin simultaneously.

For phospho-PLCγ1, blots were incubated overnight at 4 °C with rabbit polyclonal anti phospho-PLCγ1 antibody (Tyr 783) (Cell Signalling) diluted 1:1000 in 2% BSA-TBST. At the same time, α-tubulin was probed with mouse monoclonal anti α-tubulin antibody (Santa Cruz Biotechnology) diluted 1:500 in 2% BSA-TBST, overnight at 4 °C. The day after, blots were washed three times for 5 min in TBST at RT. The phospho-PLCγ1 blot was probed with HRP-linked donkey monoclonal anti-rabbit antibody (GE Healthcare) diluted 1:10000 in 2% BSA-TBST and the α-tubulin blot with HRP-linked donkey monoclonal anti-mouse antibody (GE Healthcare) diluted 1:10000 in 2% BSA-TBST for one hour at RT, washed six times for 5 min in TBST and developed using ECL Prime chemiluminiscent kit (GE Healthcare). For phospho-FAK, blots were probed using a polyclonal rabbit anti p-FAK (Tyr 397) (Merck Millipore) diluted 1:500 in 2% BSA-TBST, using a HRP-linked donkey monoclonal anti-rabbit antibody (GE Healthcare) diluted 1:10000 in 2% BSA-TBST as secondary for chemiluminiscent detection and the same protocol for α-tubulin described above. Blots were then washed six times for 5 min with TBST and bands developed using ECL Prime (GE Healthcare). All blots were imaged with a Syngene PXi 5 gel documentation system (Syngene, UK) and analyzed using ImageJ.

### 2.6 Heparin II domain availability

After coating with FN, a monoclonal antibody for the FNIII_12-14_ domain (also known as Heparin II domain) was used (Santa Cruz Biotechnologies, sc-18827) in dilution 1:30 at 37 °C for 2 h. Samples were washed three times with PBS/0.5% Tween 20. An anti-mouse IgG horse radish peroxidase (HRP)-conjugated antibody (Invitrogen, 626520) was then used in dilution 1:2000 at room temperature for 1 h. After washing twice, samples were exposed to the substrate solution (R&D, DY999) for 20 min at room temperature in the dark. A stop solution (R&D, DY994) was added before reading the absorbance at 450 nm.

### 2.7 VEGF-specific immunogold reaction

Planar samples coated with FN and with and without VEGF coating were fixed with 4% formaldehyde, incubated with primary antibody against human VEGF (Santa Cruz Biotechnologies, sc-57496) diluted 1:50 in PBS for 1 h, and after three washes with 0.5% Tween 20 in PBS, goat anti-mouse secondary antibody labeled with 15 nm gold nanoparticles (Aurion 815.022) was added in dilution 1:20 in PBS and left to react for 1 h. After immunoreaction, excess secondary Ab was removed by two PBS washes; samples were fixed with 4% formaldehyde, washed three times with ultrapure water, then gently dried with nitrogen and imaged with AFM.

### 2.8 Scanning electron microscopy

Scaffold structure was characterized by scanning electron microscope (JEOL JSM-6300) using gold-sputtered samples. The working distance was fixed at 15 mm and acceleration voltage at 13 kV. SEM images were obtained in both longitudinal and transversal sections.

### 2.9 Cell cultures

HUVEC (Cellworks) were maintained in HLVEC complete medium (Cellworks). Cells with less than 10 population doublings were used for all experiments. For network formation assays, cells were seeded at 10,000 cells/cm^2^. Seeding was done on protein-functionalized and control polymer substrates, and cells were left to adhere in CO_2_ incubator at 37 °C. After 16 h, medium was removed and replaced with a fibrinogen solution (20 mg/ml) in HLVEC basal media, containing also 50 U/ml of thrombin and 1.2 mg/ml of aprotinin. To allow complete fibrinogen clotting, samples were placed in CO_2_ incubator for 1 h. After clotting, fibrin matrix was covered with 0.5 ml HLVEC complete media with 25 ng/ml of VEGF where required, and samples were kept at 37 °C for 6 day period, with media changed every second day. For phosphorylation experiments, cells were seeded at density 15,000 cells/cm^2^ on protein-functionalized and control polymer substrates, and incubated in CO_2_ incubator at 37 °C. After 30 and 180 min, respectively, cells were washed with PBS, and then incubated with lysis buffer for 15 min on ice. Cell lysates were harvested and stored at −80 °C until assayed. For colocalization experiment, cells were seeded on control and protein-functionalized substrates at the density 6000 cells/cm^2^, and incubated in growth medium for 24 h.

### 2.10 Murine fat pad vascularization model

All animal experiments were performed in accordance with the Georgia Institute of Technology's Animal Care and Use Committee. Male mice (8 weeks old C57BL/6J mice, Jackson Laboratory) were anesthetised with isoflurane and one dose of sustained-release buprenorphine (1 mg/kg, IP) was given to provide 72 continuous hours of pain relief. The hair on the abdomen area was removed and skin disinfected with alcohol and chlorhexidine. A 10 mm midline incision was performed at the mid abdomen. The epididymal fat pad (EFP) was gently exposed and spread. One scaffold was implanted per EFP site, right and left. PMA or PEA scaffolds, functionalized with FN and VEGF, were placed on the centre on the EFP, then wrapped using the EFP tissue and sealed using a poly(ethylene glycol) sealant. The abdominal incision was closed by using a simple interrupted suture pattern with degradable suture, while the skin layer was closed with wound clips. At the endpoint of the study, 14 days post-transplant, mice were anesthetized with isoflurane, and 200 μl of DyLight 488 labeled Lycopersicon Esculentum (tomato) lectin (Vector Laboratories) in sterile saline was injected into the jugular vein with an insulin syringe. The lectin was allowed to circulate for 5 min under anaesthesia. Perfusion was performed at room temperature. After the lectin was bound to the endothelium, the abdominal cavity was opened with a midline incision and the original implant site. The vena cava was severed and the circulation flushed with saline to eliminate blood and excess lectin from the circulation, necessary for imaging purposes. All grafts were explanted, formalin fixed, paraffin embedded, and sectioned using a microtome.

### 2.11 Fluorescence staining and imaging

Cells from *in vitro* assays were washed with PBS, and then were fixed with 4% formaldehyde and permeabilized with 0.1% Triton X-100 in PBS. For network formation assay, cytoskeleton was labeled with BODIPY-FL Phallacidine (Life Technologies) diluted 1:100 in PBS, and nuclei were stained with Hoechst 33342 (NucBlue, Life Technologies) using 1 drop per 0.5 ml PBS. Images were taken with fluorescence microscope Zeiss Observer Z1 using green and blue filters; 5 areas were imaged per each sample. For phosphorylated VEGF receptor (VEGFR-2) staining, rabbit monoclonal antibody (Cell Signalling, VEGFR-2 Tyr1175) was used in 1:100 dilution and was visualized with rhodamine donkey anti-rabbit IgG (Santa Cruz Biotechnologies, sc-2095). For integrin – VEGFR-2 colocalization study, integrin α_v_/CD51 goat polyclonal antibody (R&D Systems), and VEGFR-2 rabbit monoclonal antibody (Cell Signalling) were used in combination with Alexa Fluor 546 donkey anti-goat and Alexa Fluor 647 chicken anti-rabbit secondary antibodies (Life Technologies); samples were mounted with Vectashield with DAPI (Vector Labs) and imaged using blue, red and far red filter.

Paraffin sections from *in vivo* experiment were first imaged using green filter to analyze intact fluorescence from vascular structures in the scaffolds pores; then sections were deparaffinized, stained for actin with Rhodamine Phalloidin (Life Technologies), mounted with Vectashield with DAPI to stain the nuclei, and finally imaged using red, green and blue filter as well as bright field.

### 2.12 Data analysis

Image analysis for HUVEC alignment was done in ImageJ using object counting, and area and length measurement algorithms (details in Suppl. Fig. S1). Ten areas were analyzed for each sample, and means and standard deviations were calculated. For quantification of lectin fluorescence in individual pores in scaffold sections selected areas of interest were calculated in ImageJ; in total, 30 pores were analyzed per condition. For ELISA, samples were assayed in triplicates, and means and standard deviations were calculated. For statistical analysis, either one-way ANOVA with Tukey post-test or unpaired two-tailed *t*-test was performed where applicable using GraphPad Prism5 software.

## 3 Results

### 3.1 VEGF binding

**Fig. 2:**
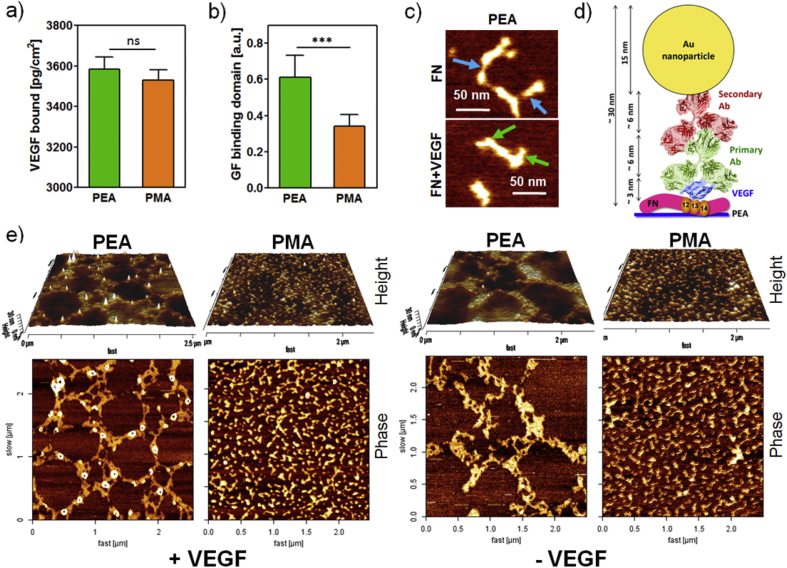
Characterization of VEGF binding to FN-coated PEA and PMA: a) VEGF bound to FN-coated PEA and PMA substrates assessed by ELISA. b) Availability of GF binding domains of FN was higher on PEA than on PMA c) AFM images of FN-coated PEA incubated with and without VEGF - images show stretched FN molecules (with monomer length ∼ 50 nm). Blue arrows depict approximate position of FNIII_12-14_ domains (GF binding site) with no GF present, green arrows show thickening of FNIII_12-14_ domains, suggesting presence of VEGF molecules. d) Scheme of immunogold binding to VEGF immobilized on surface. An expected structure height was estimated based on PDB structures 2VPF and 1IGY used for VEGF and IgG representation, respectively; structures were processed in PyMol (scheme not to scale). e) AFM imaging of FN-coated substrates after immunogold reaction with VEGF in presence of the GF (left) and without (right). White peaks represent VEGF bound to PEA whereas no VEGF was detected on PMA (left). Only FN network (PEA) or scattered FN molecules (PMA) are visible on VEGF negative controls (right). Unpaired two-tailed *t*-test was performed for statistical analysis; ***P < 0.001; ns = non-significant. (For interpretation of the references to colour in this figure legend, the reader is referred to the web version of this article.)

We quantified the total amount of VEGF bound to PEA+FN and PMA+FN surfaces as well as the availability of GF-binding domain (FNIII_12-14_) using ELISA (Fig. 2a and b). Although the surface density of VEGF on FN nanonetworks on PEA was slightly elevated in comparison to VEGF amount on FN-coated PMA, the difference was not significant (Fig. 2a). On the other hand, we observed significantly higher availability of GF binding domain for FN (FNIII_12-14_) on PEA in comparison to PMA (Fig. 2b), which should favor specific VEGF binding on fibrillar FN on PEA compared to globular FN on PMA. (Please note that we'll use the term globular FN for molecules on PMA even if the process of adsorption might lead to partial unfolding as reflected in AFM images shown in Fig. 2e).

We confirmed that VEGF was specifically bound to PEA+FN using AFM. Firstly, we hypothesized that considering the size of VEGF and that FN on PEA is organized into nanonetworks [21], VEGF-FN structures on PEA+FN would be detectable by scanning the surface with AFM. The presence of VEGF on FN fibers was difficult to observe by AFM when VEGF was adsorbed on PEA+FN compared to the control (FN-coated PEA without VEGF, Fig. 2c). Therefore, to confirm VEGF binding and specificity on FN fibrils, we used an antibody against VEGF and then a secondary antibody labeled with a 15 nm gold nanoparticle which is easily visualized by AFM. We expected an approximate height of the protein-nanoparticle complex bound to the FN-coated surface to be ∼30 nm (Fig. 2d). Fig. 2e shows that after VEGF coating, structures of the expected height were present only on FN-coated PEA samples, while FN-coated PMA samples did not show any structures apart from globular FN. No nanogold could be observed on PEA and PMA controls without VEGF coating. This result confirms that VEGF binds to FN nanonetworks organized on PEA and that VEGF binding to FN does not occur when FN adsorbed in a globular-like conformation, such as the FN-coated PMA control. Together, Fig. 2 indicates that VEGF binds specifically to FN assembled on PEA. Fig. 2e shows immunogold images, with samples under extensive washing during the incubation of antibodies that results in removal of all nanogold particles with lack of specific interactions between antibodies and VEGF. In contrast to this, Fig. 2a shows quantification of VEGF after adsorption and standard washing on the polymers, as it was done before cell culture.

### 3.2 HUVEC organization

**Fig. 3:**
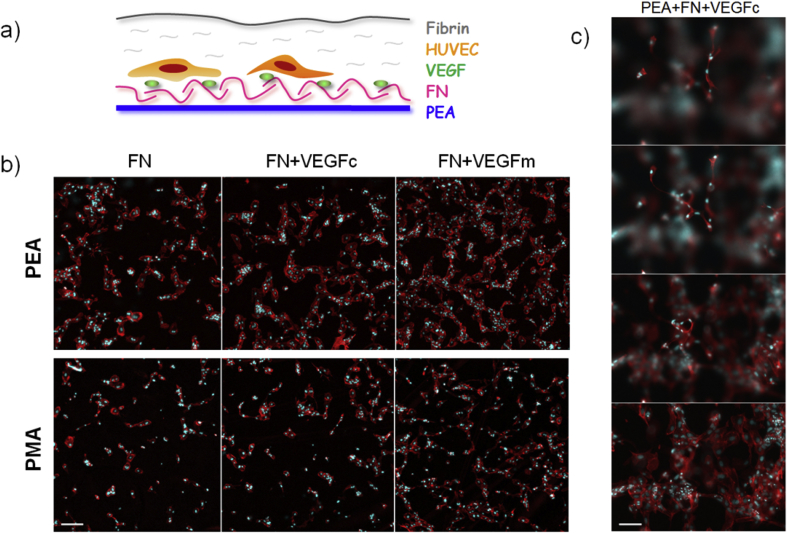
HUVEC forming network structures on functionalized substrates: a) Scheme of system components: After polymers were coated with FN and VEGF, cells were seeded on the top and finally covered with a thin layer of fibrin matrix. b) Fluorescence images of cell cultures after 6 days: FN+VEGFc coated PEA showed higher degree of aligned structures in comparison to PMA (central images), negative controls (samples with no GF coating) and positive controls (samples with no GF coating but with VEGF constantly present in media) are also shown (left and right, resp.); scale bar represents 200 μm c) Stack images showing 3D sprouting of HUVEC cells into the fibrin matrix on PEA+FN+VEGFc: bottom image is at the level of synthetic polymer substrate, height difference between bottom and top image is 200 μm; scale bar represents 100 μm.

We evaluated the ability of VEGF bound on FN nanonetworks, (i.e. VEGF as a coating: PEA+FN+VEGFc) to induce the organization of endothelial cells into tubular structures, compared to VEGF exposed to globular FN on PMA (PMA+FN+VEGFc) as well as FN nanonetworks on PEA with soluble VEGF in the culture medium (PEA+FN+VEGFm, using higher concentration of VEGF – positive control). HUVEC were seeded onto material surfaces. After cell attachment, cultures were covered with a fibrin matrix, and finally the whole construct was immersed in growth media (Fig. 3a). In this system, cells adhered ventrally to the FN+VEGF functionalized polymer surface whereas the dorsal top layer provided a supporting 3D environment representing a simplified extracellular matrix into which cell networks could form.

After 6 days of incubation, cells seeded on PEA+FN+VEGFc showed clear network alignment on the level of the polymer surface, whereas cells on PMA+FN+VEGFc exhibited considerably less developed cellular networks (Fig. 3b). For the positive control, it is noteworthy that fresh VEGF was included each time the medium was changed (Fig. 3b, FN+VEGFm) – the total amount of soluble VEGF used was ∼6 times higher than VEGF adsorbed on the surfaces. Notwithstanding this large difference in the amount of VEGF used, cell sprouting into the overlying fibrin matrix was observed for cells growing on PEA+FN+VEGFc (coating) (Fig. 3c).

**Fig. 4:**
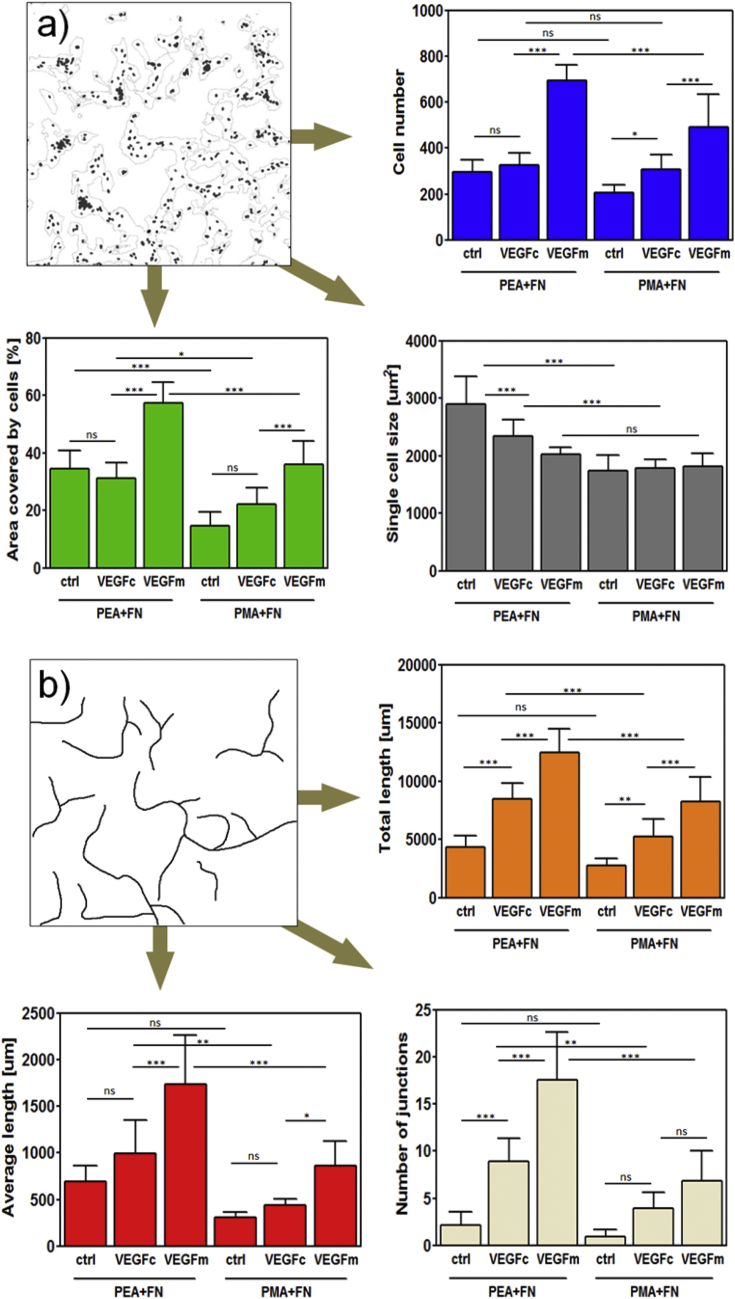
Image analysis of HUVEC behavior on functionalized substrates: a) A merged image of DAPI staining and a mask from actin staining; these raw images were used for quantification of cell count and cell spreading after 6 days of culture. The cell number (blue bars) did not vary apart from FN+VEGFm controls (FN-coated polymer substrates with VEGF present in medium). The total area coverage (green bars) was higher on PEA when compared to PMA for individual conditions: FN coated only (FN), FN and VEGF coated (FN+VEGFc), and FN-coated with VEGF in medium (FN+VEGFm), revealing better spreading on PEA-FN surfaces. This is supported by larger single cell area (grey bars) on PEA samples when comparing PEA+FN vs. PMA+FN and also PEA+FN+VEGFc vs. PMA+FN+VEGFc. b) Simplified binary image of actin staining used for quantification of cell organization; statistical analysis of total length of aligned structures per image (orange bars) as well as an average length of these structures (red bars) including number of junctions per image (cream bars) showed higher level of cell alignment on PEA+FN+VEGF samples when compared to their respective PMA+FN+VEGF controls. Total length and number of junctions were significantly higher in PEA+FN+VEGFc samples when compared to PEA+FN control, which clearly proved vasculogenic effect of VEGF coating. One way ANOVA with Tukey's multiple comparison post-test was done for statistical analysis; *P < 0.05; **P < 0.01; ***P < 0.001; ns = non-significant. (For interpretation of the references to colour in this figure legend, the reader is referred to the web version of this article.)

Quantification through image analysis of these cellular networks at the material interface revealed significant differences among groups (Fig. 4). The total cell number was similar between PEA and PMA, the same with both no VEGF and VEGF on the coating (i.e., PEA+FN, PEA+FN+VEGFc, PMA+FN and PMA+FN+VEGFc) but was increased for VEGF in the culture medium (e.g. PEA+FN+VEGFm and PMA+FN+VEGFm) (Fig. 4a, blue). However, cell attachment and spreading was better developed on PEA+FN surfaces (+/− VEGFc) when compared to PMA+FN surfaces (+/− VEGFc) as shown by the total area coverage; also PMA+FN+VEGFm had much weaker effect on coverage in comparison to PEA+FN+VEGFm (Fig. 4a green). This was also supported by larger single cell area on PEA+FN surfaces (+/− VEGFc) compared to PMA+FN surfaces (+/− VEGFc) (Fig. 4a, grey). Interestingly, the cell size showed decreasing trend on PEA when going from FN across FN+VEGFc to FN+VEGFm, with PEA+FN+VEGFm being not significantly different from all PMA samples (Fig. 4a, grey). This can be indicative of cell reorganization towards networks triggered by VEGF and then less space is needed per a single cell.

Cell alignment was quantified by measuring total length of aligned structures, average length of these structures and the number of junctions per image (Fig. 4b). All these parameters were higher for cells on PEA surfaces with VEGF (PEA+FN+VEGFc and PEA+FN+VEGFm) than on corresponding PMA surfaces (Fig. 4b). For both PEA and PMA a stepwise increase was observed from polymer+FN to polymer+FN+VEGFc to polymer+FN+VEGFm (Fig. 4b). Note that the number of cells on both PEA and PMA surfaces was similar (Fig. 4a). However the total length of aligned structures formed on PEA was higher compared to PMA (polymer+FN+VEGFc) as the total length of structures is dictated by the alignment of the cells for the same number of cells.

For conditions with VEGFm, the three parameters in Fig. 4b were still higher on PEA than PMA whereas it is notable that there were no statistical differences in total length and number of junctions between PEA+FN and PMA+FN in the absence of VEGF. This suggests that VEGF in the media might be adsorbed on the FN coated polymers as well as being free in the media and thus VEGF would promote cell organization more effectively being presented from PEA+FN as well as in the media. Overall, this quantitative analysis indicates that VEGF bound to FN nanonetworks on PEA has a positive effect in HUVECs reorganization into aligned structures.

### 3.3 Effect of RGD → RGE mutation on HUVEC organization

**Fig. 5:**
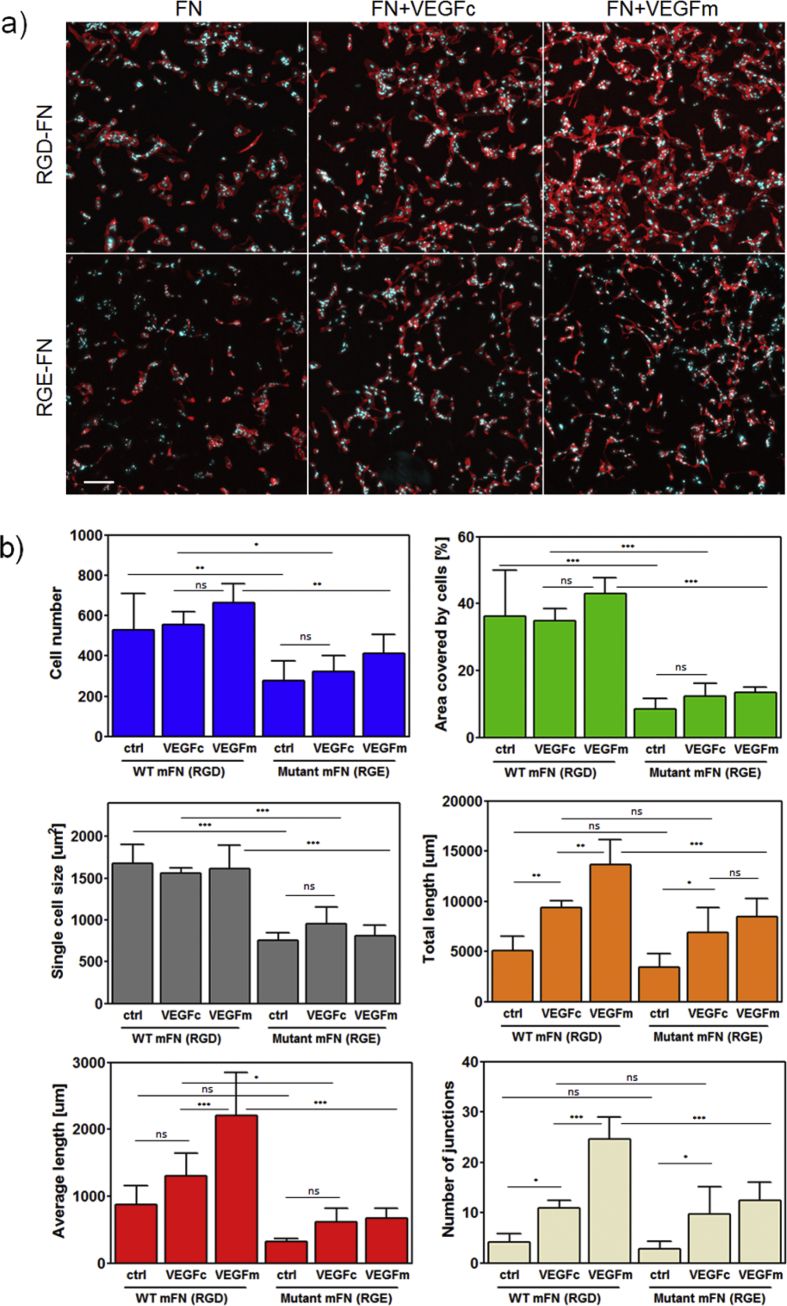
Effect of fibronectin RGD → RGE mutation on HUVEC behavior on PEA. a) Representative fluorescence images of HUVECs cultures after 6 days of incubation showed lower cell attachment and spreading on mFN-RGE coated surfaces when compared to mFN-WT (RGD). Scale bar represents 200 μm b) Image analysis of parameters characterizing cell attachment and formation of aligned structures revealed that the mutated mFN-RGE significantly decreased HUVEC numbers and spreading on PEA surfaces when compared to mFN-WT; this was valid also in presence of VEGF for both VEGF in coating (VEGFc) and VEGF in media (VEGFm) (blue, green and grey bar graphs). mFN-RGE also impaired the network formation in comparison to mFN-WT, in VEGFm samples (yellow, red and cream bar graphs), and partially in VEGFc samples (red bar graph). For statistical evaluation, one way ANOVA with Tukey's multiple comparison post-test was performed; *P < 0.05; **P < 0.01; ***P < 0.001; ns = non-significant. (For interpretation of the references to colour in this figure legend, the reader is referred to the web version of this article.)

The RGD motif in the FNIII_10_ domain is involved in integrin binding to FN. A mutant FN containing RGE (mFN-RGE) that significantly impairs cell adhesion [26] was engineered and used here to examine the role of integrin binding in the process of HUVEC organization on FN nanonetworks on PEA. We first confirmed that mFN-RGE is organized into nanonetworks on PEA, which suggests that, as expected, the RGD sequence itself is not involved in the process of PEA-driven FN assembly (Suppl. Fig. S2) [21]. Wild-type plasma FN from mouse (mFN-WT) showed comparable results to human FN in HUVEC organization experiments (Suppl. Fig. S3). In experiments where HUVEC responses were monitored on PEA+mFN-WT and PEA+mFN-RGE (Fig. 5a), cell attachment was significantly affected as parameters such as cell size, cell number and area coverage were all considerably lower in all conditions using PEA+mFN-RGE in comparison to PEA+mFN-WT (Fig. 5b – blue, green and grey).

Parameters characterizing formation of cellular networks (total length of aligned structures, their average length and number of junctions) were significantly lower on PEA+mFN-RGE with VEGFm compared to PEA+mFN-WT with VEGFm (Fig. 5b – orange, red and cream). Also for PEA+FN+VEGFc-functionalized surfaces, the average length of cellular structures was significantly lower on PEA+mFN-RGE in comparison to PEA+mFN-WT (Fig. 5b – red, compare VEGFc). Interestingly, on VEGF coated surfaces the effect of the mutation was not as obvious because the total length and number of junctions were not significantly different between PEA+mFN-RGE and PEA+mFN-WT (Fig. 5b – orange and cream, compare VEGFc). It is clear that the aligned structures on PEA+mFN-RGE with VEGF coating were shorter when compared with PEA+mFN-WT. However, cells on PEA+mFN-RGE were still able to form network structures of comparable total length and with similar number of junctions.

### 3.4 Cell signalling

**Fig. 6:**
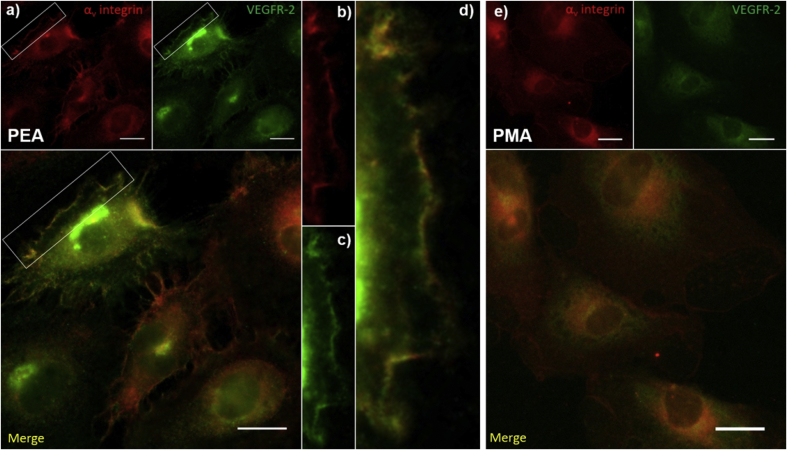
Integrin α_v_ and VEGF receptor colocalization in HUVEC on polymer+FN+VEGFc surfaces after 24 h of incubation: a) Cells on PEA coated with FN and VEGF (PEA+FN+VEGFc) stained in red for integrin α_v_ and in green for VEGFR-2; top images represent individual channels while the bottom image shows their merge; white rectangles point out example area where both proteins were detected in the same location which resulted in yellow colour in the merge image. Detailed view of this area for integrin α_v_, VEGF receptor and their merge is shown on b), c) and d), respectively; e) Cells on PMA coated with FN and VEGF (PMA+FN+VEGFc) stained in the same way as on the PEA sample with individual red and green channels at the top and merge image at the bottom; integrin α_v_ staining is present while clear VEGFR-2 staining is not obvious (the faint staining is mainly associated with background). Scale bar represents 20 μm on every image. (For interpretation of the references to colour in this figure legend, the reader is referred to the web version of this article.)

To test whether PEA-driven integrin/VEGF presentation drives HUVEC organization through a synergistic mechanism, we performed co-localisation and signalling experiments. VEGF was adsorbed onto FN-coated PEA and PMA surfaces and then we examined colocalization of integrins and VEGFR-2 at the individual cell level using immunofluorescence (Fig. 6). We observe colocalization of α_v_ and VEGFR-2 (but not of β_1_ results not shown) on PEA+FN+VEGFc (Fig. 6a–d). There is no colocalization of receptors on PMA (Fig. 6e) which supports the idea that VEGF bound next to the RGD sequence facilitates simultaneous VEGFR-2 and integrin α_v_ binding within the same nanoscale cluster.

We assessed VEGF receptor (VEGFR-2) phosphorylation as a direct measure of VEGF signal activation. Immunofluorescence for phosphorylated VEGFR-2 was performed on HUVEC cultured on tissue culture plastic, after cells were stimulated with 25 ng/ml of VEGF for two different time periods (2 and 30 min). After 2 min of stimulation, the phosphorylated receptor was observed while after 30 min incubation no phosphorylation was detected (Suppl. Fig. S4) [27]. This result showed that the time window for VEGFR-2 phosphorylation is very narrow and so detection of VEGFR-2 phosphorylation in response to the surfaces challenging considering that cells require some time to adhere. To investigate signalling, we examined phosphorylation of phospholipase C gamma 1 (pPLCγ1) as a downstream effector which is activated by VEGFR-2 [4], phosphorylation of focal adhesion kinease (pFAK) that is activated after integrin binding and clustering [28], and phosphorylation of ERK1/2 (pERK1/2) a downstream effector which is activated via the mitogen activated protein kinase (MAPK) branch of GF receptor and integrin signalling [4].

**Fig. 7:**
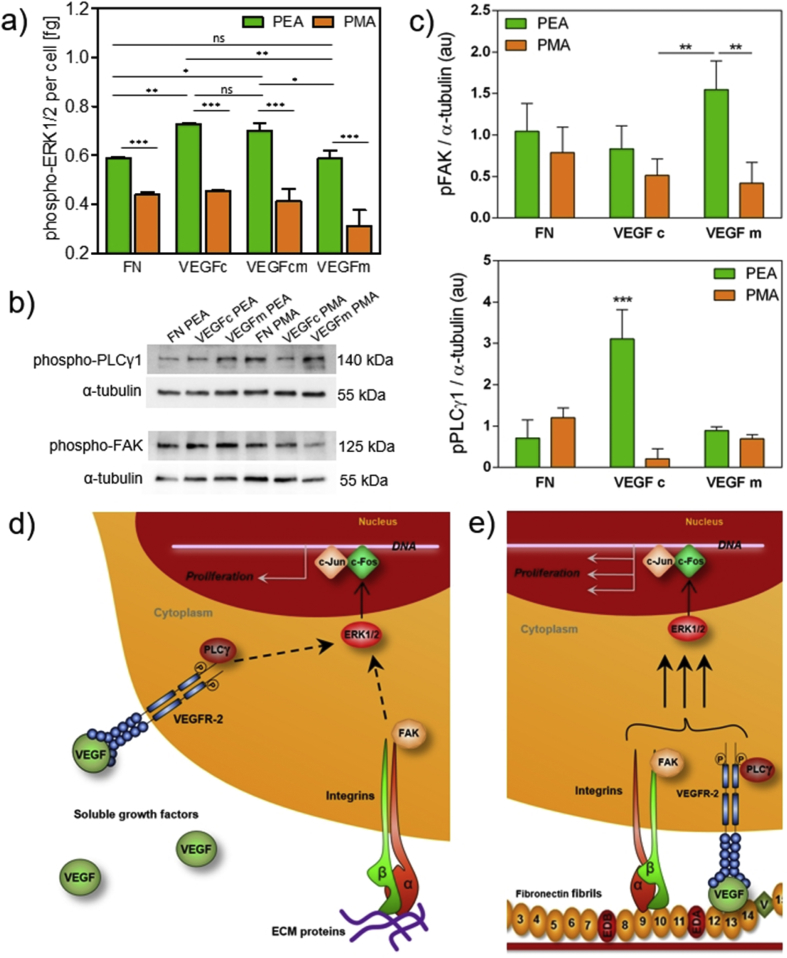
ERK1/2, PLCγ1 and FAK phosphorylation in HUVEC on PEA and PMA coated with fibronectin: a) ELISA quantification of pERK1/2 after 30 min incubation showed significantly higher phosphorylation in cells on PEA (green bars) than on PMA (orange bars). On PEA+FN substrates, VEGF-coated sample (VEGFc) showed higher level of ERK1/2 phosphorylation when compared to PEA+FN without VEGF (FN). PEA+FN sample with VEGF present in both coating and media (VEGFcm) showed no difference from PEA+FN+VEGFc, and PEA+FN with VEGF in media only (VEGFm) did not vary from PEA+FN control. b) Representative images of western blot membranes with detected phospho-PLCγ1 and phospho-FAK proteins in HUVEC lysates after 30 min and 2 h incubation, respectively; bands were normalized against α-tubulin; c) Quantification of phosphorylated proteins from phospho-PLCγ1 and phospho-FAK western blot bands. Enhanced VEGF signalling is observed on PEA+FN+VEGFc d) Scheme of individual VEGFR-2 and integrin signalling pathways towards ERK1/2 stimulation depicting the role of PLCγ1 and FAK as early effectors of VEGFR-2 and integrin signal transduction; phosphorylated mitogen-activated kinase 1 and 2 (ERK1/2) leads to activation of the c-Fos transcription factor. Its formation of heterodimers with c-Jun and binding to DNA. e) Scheme of synergistic VEGFR-2 and integrin signalling towards pERK1/2 can lead to enhanced ERK1/2 stimulation. For statistical evaluation, one way ANOVA with Tukey's multiple comparison post-test was performed; *P < 0.05; **P < 0.01; ***P < 0.001; ns = non-significant. (For interpretation of the references to colour in this figure legend, the reader is referred to the web version of this article.)

No differences were observed in pFAK when no VEGF or VEGF was used as a coating on both PEA and PMA. pFAK was significantly higher on PEA when VEGF was added in the medium, which suggests that soluble VEGF reinforces adhesion to FN nanofibrils but do not VEGF signalling (Fig. 7c). However, after 30 min of adhesion, significantly higher phosphorylation of PLCγ1 and ERK1/2 was observed on PEA+FN+VEGFc compared to the other conditions (no VEGF and VEGF in the medium for both PEA and PMA, Fig. 7a–c). We note that higher ERK1/2 phosphorylation on PEA+FN and PEA+FN+VEGFc compared to the corresponding PMA surfaces was not translated into a higher level of cell proliferation (similar number of cells show on Fig. 4a) but must have played role in the ability of cells to migrate and organize on the surfaces [29].

Importantly, differences were found between PEA based samples themselves for VEGF related signalling. FN nanonetworks on PEA coated with VEGF (PEA+FN+VEGFc in Fig. 7) showed a higher level of both PLCγ1 and ERK1/2 phosphorylation in comparison with PEA+FN with no VEGF and PEA+FN+VEGFm (Fig. 7a–c). Interestingly, ERK1/2 phosphorylation on PEA+FN shows no difference for VEGFc and VEGF that has been both coated and added to the media (PEA+FN+VEGFc and PEA+FN+VEGFcm in Fig. 7a), i.e. there is no additional ERK1/2 phosphorylation related to the presence of VEGF in the medium when the GF is already on the coating. It is further noteworthy that the lack of effect of VEGF in the medium on pERK1/2 is supported by similar levels on PEA+FN+VEGFm over PEA+FN (Fig. 6a).

In summary, these results confirm enhanced effects of FN bound ligands, as only the bound VEGF in close vicinity to integrin binding sites was able to stimulate cells at the highest level – i.e. PEA where VEGF was bound on FN nanonetworks in synergy with integrins. Soluble VEGF in the medium did not have any significant effect at this early time point.

### 3.5 *In vivo* vascularization

**Fig. 8:**
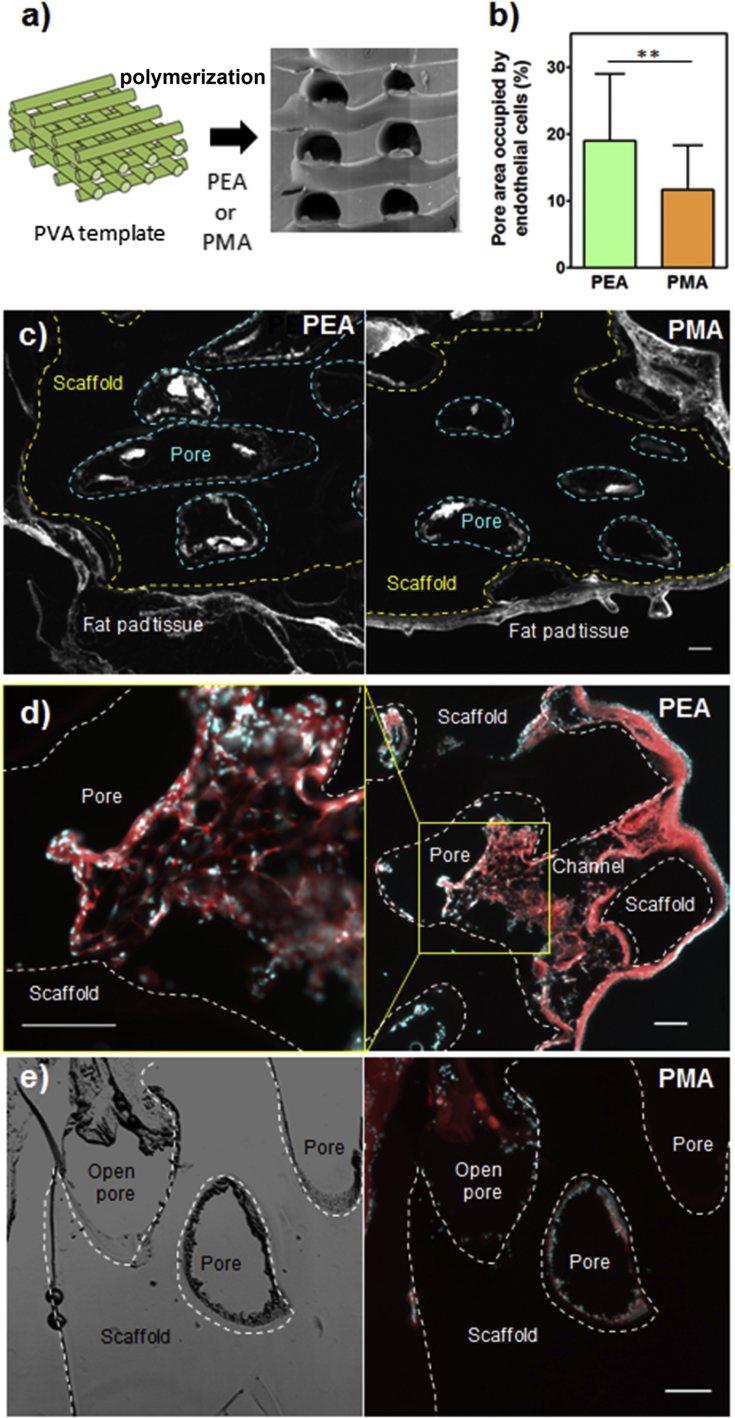
Murine fat pad model for vascularization showed better response for PEA than for PMA scaffolds; a) Fabrication of scaffolds: PEA and PMA was polymerized in 3D printed PVA templates to form scaffold with interconnected channels. b) Quantification of lectin fluorescence representing endothelial cells infiltrating PEA+FN+VEGFc and PMA+FN+VEGFc (**P < 0.01). c) Representative images of thin sections of explanted PEA and PMA scaffolds show endothelial cell specific lectin fluorescence staining of original fat pad tissue as well as new endothelial cells inside the pores; fluorescence inside the pores was used for quantification. Limits between scaffolds and fat pad tissue are shown by the yellow dashed line; pores within the scaffold are delimited by blue dashed lines. Fat pad tissue surrounds completely both PMA and PEA scaffods. d) Detailed image of newly formed tissue inside a pore of the PEA+FN+VEGF scaffold showing vascular network; lower magnification image on the right shows position of the pore in the scaffold and link to the original fat pad tissue through a channel. e) Lack of formation of tissue inside pores of the PMA+FN+VEGF scaffolds together with the corresponding bright field image that has allowed univocal identification of pores. The corresponding bright field image for PEA is include in Supplementary Fig S5e. Cytoskeleton is in red, nuclei in cyan, scale bars represent 100 μm. (For interpretation of the references to colour in this figure legend, the reader is referred to the web version of this article.)

To test whether our system can stimulate vascularization *in vivo*, we fabricated PEA and PMA scaffolds using a 3D printed template (Fig. 8a, Suppl. Fig. S5a, S5b). Experiments with murine mesenchymal stem cells (MSCs) demonstrated robust cell adhesion and scaffold infiltration (Suppl. Fig. S5c). For *in vivo* studies, PEA and PMA scaffolds were coated with FN and VEGF, and implanted in murine epididymal fat pads. After two weeks, mice were perfused with a solution of fluorescent lectin which binds to endothelial cells in functional vasculature. After fixing and sectioning, lectin fluorescence was quantified inside the scaffold pores. Significantly higher fluorescence was detected inside the pores for PEA+FN+VEGFc scaffolds in comparison to PMA+FN+VEGFc scaffolds used as a control (Fig. 8b), showing PEA+FN+VEGFc promoted enhanced levels of vascularization.

Sections were also stained for cytoskeleton and nuclei, and both for PEA+FN+VEGFc and PMA+FN+VEGFc, a thin layer of the original fat pad tissue was detected around the scaffold. Also, cell ingrowth was present in open pores or in pores directly connected with the original tissue through channels in both PEA+FN+VEGFc and PMA+FN+VEGFc implants. However, PEA+FN+VEGFc scaffolds resulted in higher level of vascularization with newly formed vessels clearly visible (Fig. 8e, Suppl. Fig. S5e).

## 4 Discussion

Blood supply is essential for successful tissue engineering strategies with poor supply leading to ischemic conditions such as in diabetic wounds and ulcers [30]. The only GF-based product approved for the treatment of complex wound healing (Regranex^©^) contains platelet-derived growth factor-BB (PDGF-BB) and has limited efficacy along with safety concerns (risk of cancer warned by FDA in 2008). These concerns result from spatiotemporally uncontrolled and supraphysiological dosing (100 μg/g) to compensate for clearance from the damaged area.

Clinical trials with VEGF delivery for cardiac repair have not been successful either, failing to demonstrate significant improvement in patients when compared to placebo [31]. As well as for cardiac application [32], therapeutic VEGF delivery has been studied for wound healing (reviewed in Ref. [33]) or bone repair (reviewed in Ref. [34]). However, overall VEGF use in pro-angiogenic therapies has not been effective thus far [35], [36]. Again, use of high GF dose severely limits translation of VEGF use into clinical practice.

Therefore, there is much interest in the development of pro-angiogenic biomaterials that present rather than deliver GFs so that low doses can be used locally without systemic collateral damage [8], [37]. A number of design criteria have emerged over the years, which combine the use of microenvironments that recapitulate the physiological ECM, in particular the presence of defined cell adhesion sites [38] and the ability to bind angiogenic factors and stimulate GF receptors [12], [27], [38].

A particularly efficient approach has been developed using FN domains containing both cell attachment and GF binding motifs (FNIII_9-10_/FNIII_12-14_) into fibrin matrices to promote synergistic integrin/GF receptor signalling [27]. However, this approach relies on recombinant FN fragments and soft materials.

We have used a material system (PEA) that promotes the self-organization of FN to present cell adhesion- and GF-binding domains that sequester GFs including VEGF [18], [22]. FN nanonetworks on PEA promoted cell adhesion and maintenance of stem cell phenotypes in the absence of GFs [20], [39], [40], [41]. PEA driven FN nanonetworks allow efficient GF presentation and we have shown that PEA+FN+BMP-2 increased osteogenic differentiation in MSCs compared with soluble BMP-2 along with bone regeneration in a critical size defect, [22].

Here, we used PEA to assemble FN molecules and bind VEGF seeking to promote simultaneous VEGFR-2 and integrin signalling (Fig. 7e). We found VEGF interactions with FN molecules exclusively on PEA but not on PMA (where FN remains globular) (Fig. 2), and that in both *in vitro* (Fig. 3) and *in vivo* (Fig. 8) tests the vasculogenic response was significantly enhanced on VEGF-FN-coated PEA due to efficient VEGF binding to FN, which has been reported to occur on FNIII_12-14_ in close vicinity of FN integrin binding domains [18]. This was achieved with an ultra low dose of GF (as a single dose) (∼5 ng/cm^2^), which represents a significant advantage of this system over other strategies used so far [13], [42], [43].

While VEGF has been incorporated into material systems as it regulates cell migration and proliferation of existing ECs [4] as well as recruitment of EC-progenitors and perivascular cells to promote tissue repair [44], [45], VEGF crosstalk with integrins still has been poorly addressed in the design of material systems. We show colocalization of integrin (α_v_) and VEGFR-2 (Fig. 6). It was previously shown that phosphorylation of VEGF receptor-2 (VEGFR-2) was prolonged in association with integrins [46], and ERK1/2 (crucial for gene expression and DNA synthesis) was phosphorylated as a result of activation of both integrins and VEGFR-2 via the FAK/Ras/Raf (integrin based ERK cascade) and PLCγ/Raf (VEGFR-2 based ERK cascade) pathways respectively [47], [48]. We show enhanced PLCγ phosphorylation when VEGF is presented on PEA+FN, which reveals enhanced GF signalling in crosstalk with integrins (Fig. 7).

As the RGD sequence of FNIII_10_ binds particularly integrins α_5_β_1_ and α_v_β_3_ dimers [16], we hypothesize that these integrins cooperate with VEGFR-2 in our system (PEA+FN+VEGFc). The role of β_1_ was supported by previous results using VEGF in collagen, where integrin β_1_ recruitment and VEGFR-2 clustering was linked to increased DNA synthesis and cell migration [46]. A study using recombinant fragments of FN (FNIII_9-10_/FNIII_12-14_) in fibrin matrices demonstrated the central importance of α_5_β_1_ integrin for improved EC migration, proliferation and tube-like structure formation while α_v_β_3_ was less critical [27]. Our studies show a preference for α_v_ to colocalise with VEGFR-2 on fibrillar FN on PEA (Fig. 6). The role of integrins in VEGFR-2 mediated crosstalk remains complex and requires further elucidation. For example, α_v_β_3_ has been related to VEGF-stimulated initiation of angiogenic programing [49], [50], ECs on FN showed weaker response in comparison to vitronectin, which binds exclusively α_v_β_3_, and α_v_β_3_ (but not β_1_) was identified using a synthetic FNIII_10_-VEGF fused protein [14].

With respect to use of RGE to determine the role of RGD-mediated integrin binding, it has been reported that i) FN RGE mutations impair α_5_ binding but still allow α_v_ binding, and ii) that engineered FN-RGE mice have the same phenotype as α_5_ integrin-null mice [26]. In our study, mFN-RGE showed significantly lower support of HUVEC networks than mFN-WT (RGD) on PEA (Fig. 5), indicating a key role of integrins α_5_ and α_v_ in this process. However, at the same time, there were still a small number of cellular network structures observed in ECs on mFN-RGE (Fig. 5), which suggests that α_v_ could still bind and be involved in synergistic signalling up to a certain level. This is supported by co-localisation experiments shown in Fig. 6. Overall, our experiments with mutant FN containing RGE (mFN-RGE) suggest that both α_5_ and α_v_ integrins cooperate in HUVEC stimulation (together with VEGF) (Fig. 5).

Increased vascularization *in vivo* showed that PEA promoted presentation of VEGF is functional and that *in vitro* effects can be translated *in vivo*. A major advantage of our system is that the VEGF presentation occurs from a ‘solid-phase’ allowing enhancement through synergy with adhesion signalling [51]. The solid phase use of VEGF involves presentation rather than delivery and the synergy effect allows us to control the GF concentration; this means off target effects should be avoided. We used a solution of VEGF at a concentration 10 μg/ml to saturate all the binding sites of FN on the surface of PEA. Since the amount of FN adsorbed on these polymers is ∼400 ng/cm^2^ (from a solution of 20 μg/ml) [21], the maximum amount of VEGF that remains specifically bound on the surface is < 50 ng/cm^2^. Considering the geometry of the scaffold (a mesh of channels with diameter 400 μm separated at a distance of 300 μm), the surface to volume ratio of the pores is 100 cm^−1^, which allows to calculate the equivalent concentration of VEGF within the scaffold volume to be < 5 μg/ml. Note that this concentration is at least 50% lower than the standard amount of VEGF used in vascularization studies using advanced material systems [52], [53], [54].

## 5 Conclusions

We have engineered a system that promotes vascularization *in vitro* and *in vivo* using low doses of VEGF bound to a material surface. The system is based on a polymer (PEA) that organizes FN into nanonetworks to expose simultaneously the integrin (FNIII_9-10_) and GF (FNIII_12-14_) binding regions of FN to promote specific VEGF binding and then synergistic integrin/VEGF receptor signalling. The system is robust, simple and efficient and it has the potential to be translated as it can be incorporated (e.g. coatings) on 2D and 3D devices and scaffolds.

## Data and materials availability

All the original data related to this article are within the depository of the University of Glasgow with doi:10.5525/gla.researchdata.390. Additional data related to this paper may be requested from the authors.
