# Supplementary material for: Engineered microenvironments for synergistic VEGF – Integrin signalling during vascularization
Source: Biomaterials. 2017 May;126:61–74. doi: 10.1016/j.biomaterials.2017.02.024 (PMC5354119; doi:10.1016/j.biomaterials.2017.02.024)
Supplement: Supplementary file 1 [file mmc1.docx]

Engineered microenvironments for synergistic VEGF – integrin signalling during vascularization

Vladimíra Moulisová^1^, Cristina Gonzalez-García^1^, Marco Cantini^1^, Aleixandre Rodrigo-Navarro^1^, Jessica Weaver^5^, Mercedes Costell^2^, Roser Sabater i Serra^3^, Matthew J. Dalby^4^, Andrés J. García^5^ and Manuel Salmerón-Sánchez^1^

*^1^Division of Biomedical Engineering, School of Engineering, University of Glasgow, Glasgow G12 8LT, United Kingdom*

*^2^ Departament de Bioquimica i Biologia Molecular, Universitat de València, Burjassot, Spain*

*^3^ Centre for Biomaterials and Tissue Engineering. Universitat Politècnica de València, Spain & Networking Research Center on Bioengineering, Biomaterials and Nanomedicine (CIBER-BBN), Valencia, Spain*

*^4^ Center for Cell Engineering, Institute of Molecular Cell and Systems Biology, University of Glasgow, Joseph Black Bld, University Avenue, Glasgow G12 8QQ, UK.*

*^5^ Woodruff School of Mechanical Engineering, Petit Institute for Bioengineering and Bioscience, Georgia Institute of Technology, Atlanta, GA 30332, USA*

**Supplementary material**

| \| 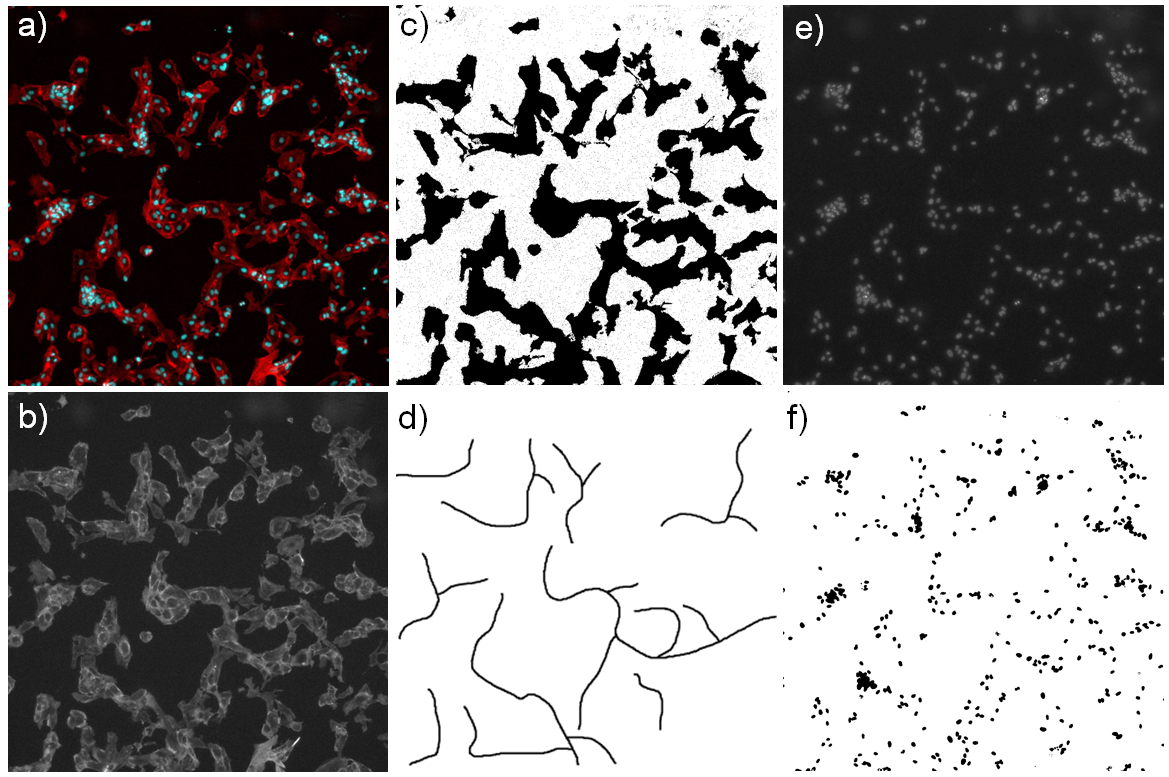  **Supplementary figure S1.** Example of image processing used for image analysis of HUVEC growth and alignment on functionalized substrates (PEA-FN-VEGF-coated example): a) composite image of actin (red) and DAPI (cyan) staining; b) raw image of actin staining from which a mask image (c) was prepared and used for quantification of cell area coverage. Raw actin image was also used for making a simplified binary image cell alignment (d) from which lengths and numbers of aligned structures and junctions were calculated; e) raw image of DAPI staining from which a binary image was produced to count cells per area. \| \| --- \|   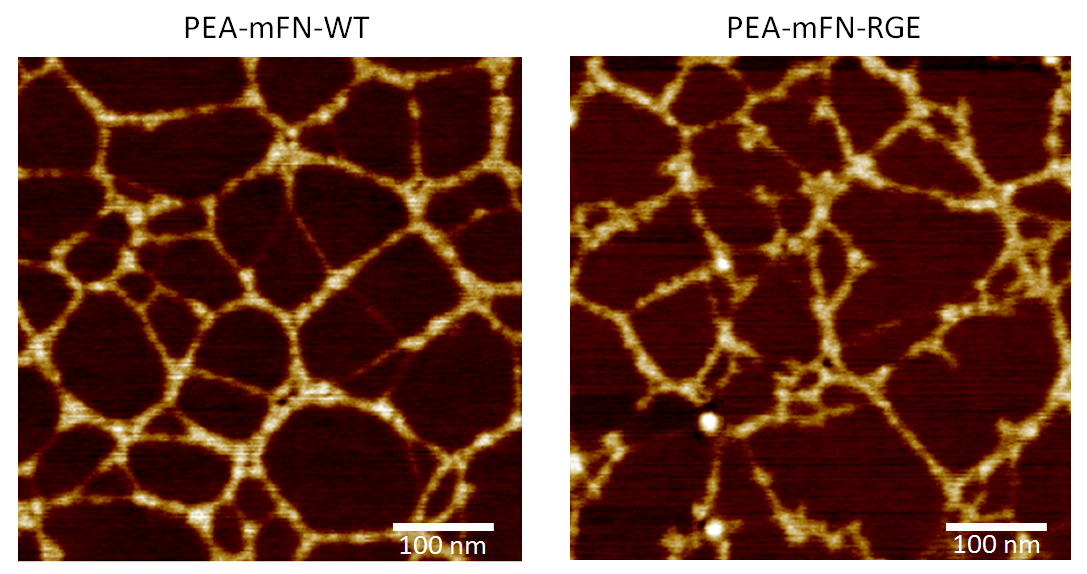  **Supplementary figure S2.** Visualization of FN molecules on PEA surface; wild-type mouse FN with RGD motif (mFN-WT) and mutant mouse FN with RGE motif (mFN-RGE); the material triggered FN fibrillogenesis appeared in both cases confirming the D 🡪 E mutation in FN had no effect on this process. |
| --- | --- |
| 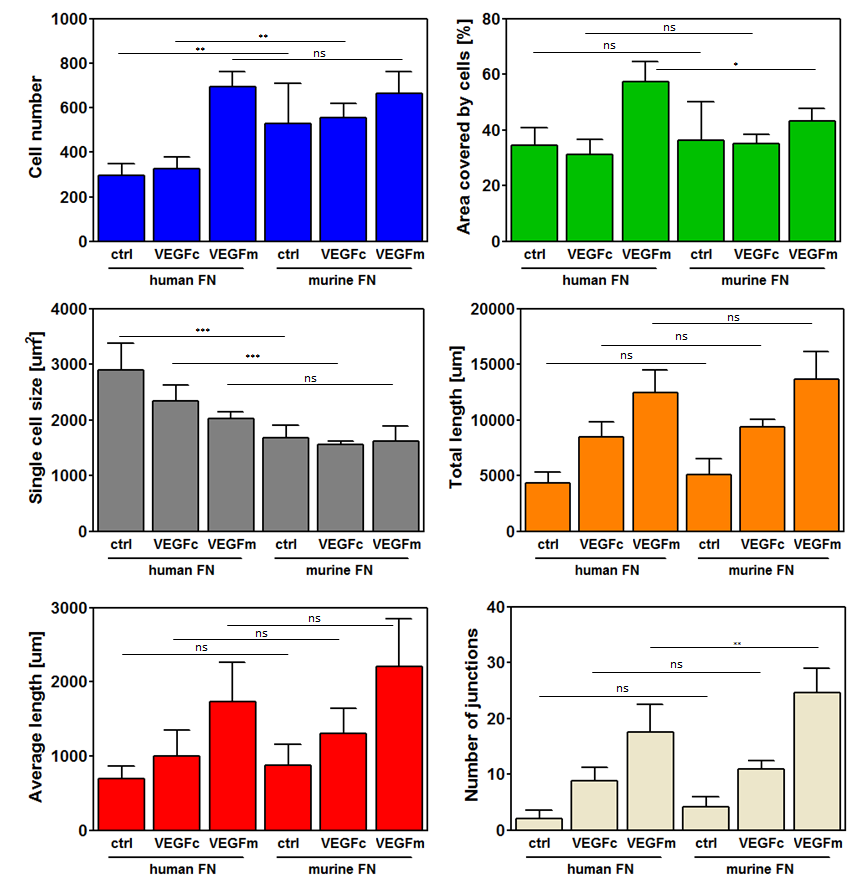 |
| **Supplementary figure S3.** Comparison of effect of human and murine FN on HUVEC behavior; both types have similar effect; particularly in case of lengths of aligned structures (the differences between the same conditions regarding GF presence are not statistically significant). For statistical evaluation, one way ANOVA with Tukey’s multiple comparison post-test was performed; * P<0.05; ** P<0.01; *** P<0.001; ns = non-significant. |

| 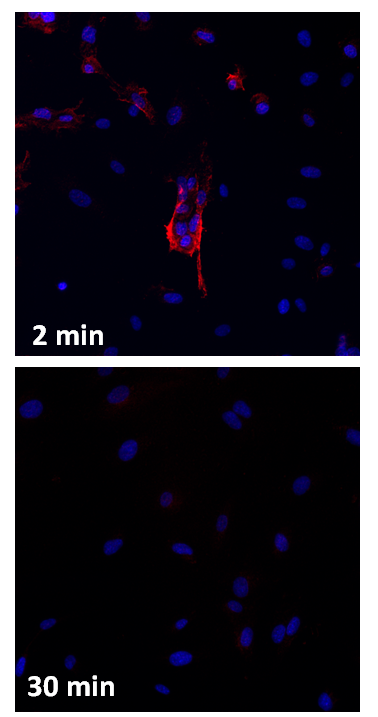 |
| --- |
| **Supplementary figure S4.** Phosphorylation of VEGFR-2 in HUVEC after 2 and 30 min of stimulation with VEGF. After 2 min of stimulation the phosphorylated receptor was observed (red staining), while after 30 min incubation no phosphorylation was detected anymore (blue staining for nuclei). |

| 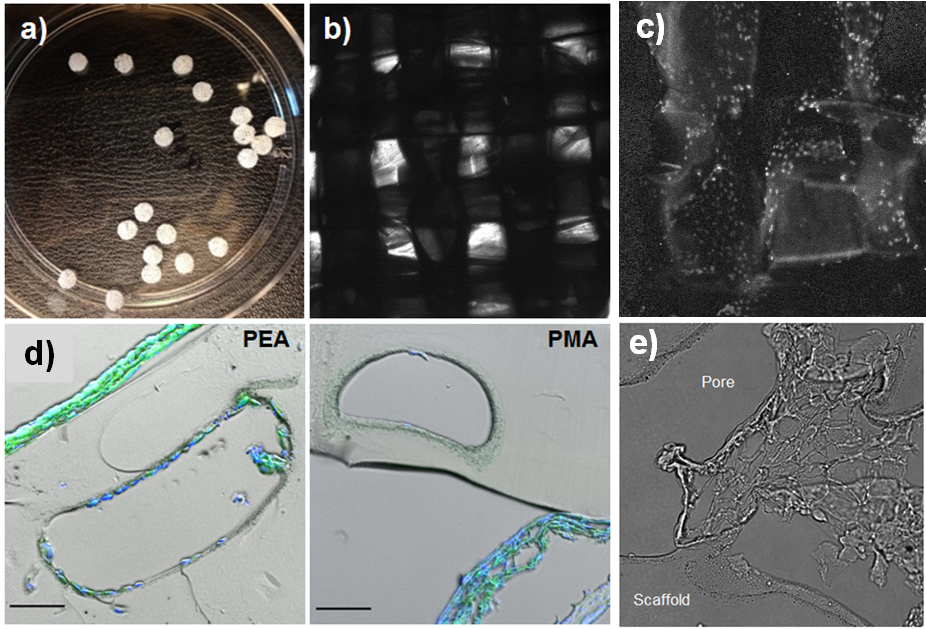 |
| --- |
| **Supplementary figure S5.** *In vivo* vascularization: a) A photograph of PEA scaffolds after polymerization; the diameter of a scaffold is 5 mm; b) Microscopic image of polymerized PEA scaffold with pores and channels; c) Fluorescence image of MSCs adhered inside the PEA scaffold (cells stained with DAPI); d) Composite images of thin sections of the explanted scaffold showing original fat pad tissue as well as cells inside the pores; green represents lectin stained endothelial cells, nuclei are blue. Scale bars represent 100 μm; e) Bright field image of newly formed tissue inside the pore of PEA scaffold that corresponds to the fluorescence image shown on Fig. 8d. |
